# La tuberculose des amygdales palatines

**Published:** 2010-01-03

**Authors:** Cherkaoui Amine, Hajjij Amal, Ouatassi Nawar, Oudidi Abdelatif, El alami Nouredine

**Affiliations:** 1 Service ORL et Chirurgie-Cervico-Facial CHU Hassan II Fes, Maroc

**Keywords:** Tuberculose, amygdale, traitement antibacillaire, tonsil, tuberculosis

## Abstract

**Introduction:**

La localisation tuberculeuse des amygdales palatines est très rare même dans un pays d’endémie tuberculeuse. Le diagnostic peut être suspecté cliniquement dans un contexte évocateur, mais il ne peut être certifié qu’au stade histologique.

**Patients et méthodes:**

Cinq cas de tuberculose des amygdales palatine ont été diagnostiqués sur une période de 5 ans et inclus dans une étude rétrospective. Nous avons recensé trois hommes et deux femmes (âge moyen de 28 ans). La dysphagie haute était le maître symptôme. L’examen a objectivé une hypertrophie amygdalienne chez tous les patients avec des ulcérations chez trois d’entre eux. Une biopsie a été effectuée chez trois patients et une amygdalectomie a été réalisée chez les deux autres. L’étude anatomopathologique a posé le diagnostic chez tous les patients. Un traitement médical antituberculeux selon un régime court allant de 6 à 9 mois a été instauré. Dans tous les cas, l’évolution à long terme a été favorable avec un recul moyen de 13 mois.

**Conclusion:**

La tuberculose amygdalienne est rare. Son diagnostic repose essentiellement sur le résultat anatomopathologique de la biopsie où de l’amygdalectomie. L’évolution sous traitement anti-bacillaire est souvent favorable, la récidive est exceptionnelle.

## Introduction

La tuberculose est une maladie qui connaît un renouveau à cause de l’émergence du syndrome d’immunodéficience acquise et de l’apparition de souches multi résistantes du bacille tuberculeux. Elle sévit à l’état endémique dans les pays en voie de développement sans toutefois épargner les pays industrialisés. Parmi les formes extra-pulmonaires, la localisation au niveau des amygdales palatines est rare et peu décrite dans la littérature. A travers cette observation, nous nous proposons de préciser les modalités diagnostiques et thérapeutiques et évolutives de cette affection.

## Patient et méthodes

De Mars 2003 à Mars 2009, cinq patients ont été diagnostiqués dans le service d’ORL CHU Hassan II de Fès (Maroc) pour tuberculose de l’amygdale palatine.

Il s’agissait de trois hommes et de deux femmes, âgés en moyenne de 28 ans (extrêmes: 15–42 ans). Un antécédent de tuberculose pulmonaire traitée a été noté chez deux malades avec une notion de contage tuberculeux chez un patient. Un bas niveau socioéconomique a été noté chez tous les malades.

## Résultats

Le délai de consultation par rapport au début de la symptomatologie était inférieur à 9 mois chez tous les patients. Les signes cliniques constants étaient: la dysphagie haute, une fièvre, une asthénie et un amaigrissement. Les signes associés étaient dominés par l’odynophagie et le mauvais état dentaire retrouvé respectivement chez trois et deux patients. L’état général était altéré chez un malade. L’examen clinique a objectivé une hypertrophie amygdalienne bilatérale chez tous les patients avec des ulcérations chez trois d’entre eux, des granulations avec un érythème non induré chez un patient et des taches blanchâtres chez un autre. Des adénopathies cervicales jugulo-carotidiennes bilatérales de petite taille et de consistance ferme ont été notée chez deux patients. Le bilan biologique comportant une numération formule sanguine et une vitesse de sédimentation était accélérée chez deux patients. L’intradermo-réaction a été positive chez 3 des patients. La sérologie VIH, faite chez deux malades est revenue négative. La radiographie thoracique réalisée pour tous les patients a objectivé des lésions tuberculeuses séquellaires chez deux d’entre eux.

Le diagnostic a été posé par l’examen histo-pathologique de la biopsie des trois patients chez qui le diagnostic a été suspecté cliniquement et de l’amygdalectomie bilatérale réalisée chez les deux autres. Un traitement médical a été instauré et consistait d’anti-bacillaires en régime court de six mois chez trois patients et de neuf mois chez les deux patients ayant des antécédents de tuberculose pulmonaire. Le protocole thérapeutique comportait : une première phase d’attaque de 2 mois associant 3 anti-bacillaires (Isoniazide (H): 5–8 mg/kg/j, Rifampicine (R): 10 mg/kg/j) et Pyrazinamide (Z): 35 mg/kg/j). Une phase d’entretien de 4 à 7 mois selon la durée du traitement avec 2 anti-bacillaires (R: 15 mg/kg/j et H: 10–15 mg/kg/j). Dans tous les cas, l’évolution à long terme a été favorable avec un recul moyen de 13 mois.

## Discussion

Décrite pour la première fois par Dieulafoy en 1895 [1], la localisation tuberculeuse des amygdales palatines est rare. Dans une étude faite par Cap’o en 1947, l’incidence moyenne de la tuberculose amygdalienne était de 2,2 pour 100000 [2].

L’introduction de la vaccination et de la chimiothérapie anti-bacillaire ainsi que la pasteurisation du lait, ont entraîné une nette régression de l’amygdalite tuberculeuse résultante de l’infection à *Mycobacterium (M) bovis* contaminant le lait de vache [3, 4]. L’atteinte amygdalienne est devenue exceptionnelle dès 1995 dans le monde occidental [5] et demeure rare malgré la recrudescence actuelle de la tuberculose redevenue un véritable problème de santé publique à l’échelle planétaire ne se limitant pas seulement aux zones à endémie élevée. Ce renouveau est dù à la dégradation des conditions socio-économiques des populations, à l’infection par le VIH et l’apparition de souches multi et ultra-résistantes de bacilles tuberculeux [6].

La tuberculose amygdalienne est le plus souvent secondaire à une localisation pulmonaire [7, 8, 9]. Elle est favorisée par l’alcoolisme chronique et par le syndrome de d’immunodéficience acquise [10]. Les caractéristiques cliniques ne sont pas spécifiques, simulant parfois le tableau d’une amygdalite chronique [11], comme dans le cas chez une de nos patientes.

En l’absence d’autres localisations évocatrices, d’une notion de contage ou d’antécédents de tuberculose, les symptômes subjectifs de la primoinfection tuberculeuse amygdalienne est faite essentiellement de dysphagie haute et/ou odynophagie [12]. L’examen peut objectiver une hypertrophie amygdalienne, des ulcérations, des granulations avec un érythème non induré, des taches blanchâtres ou jaunâtres et parfois des adénopathies latéro-cervicales essentiellement jugulo-carotidiennes [10, 13].

Le diagnostic différentiel comporte les ulcères traumatiques, les aphtes, l’angine de Vincent, les affections hématologiques, l’actinomycose, la syphilis, la granulomatose de Wegener, les carcinomes primaires et les métastases [14, 15]. Le bilan biologique peut être normal ou révéler un syndrome inflammatoire. Une intradermoréaction négative à la tuberculine ne permet pas d’écarter une étiologie tuberculeuse. La radiographie thoracique et la recherche du bacille de Koch (BK) dans les crachats ou dans les produits d’aspirations bronchiques par l’examen microbiologique classique ou par PCR (Polymerase Chain Reaction) permettent parfois de dépister une atteinte pulmonaire associée.

L’examen sérologique concernant le VIH est justifié vu la fréquence de la coexistence des deux infections VIH et tuberculose ainsi qu’une prépondérance marquée des formes extra-pulmonaires de la tuberculose chez les patients immunodéprimés [16].

Le diagnostic de certitude repose sur l’identification du BK et à l’exclusion des mycobactéries non tuberculeuses par la culture des prélèvements biopsiques [7, 10, 17]. Ce prélèvement biopsique doit être fractionné: un premier fragment, destiné à la bactériologie, est placé dans un tube sec. La culture positive apportera la preuve formelle du diagnostic de tuberculose. Un second fragment est destiné à l’examen anatomo-pathologique [18].

Le diagnostic bactériologique repose sur la mise en évidence des bacilles tuberculeux, à savoir *M tuberculosis*, le plus fréquent, *M bovis* ou *M africanum*, ces trois espèces très proches étant réunies sous le nom de complex tuberculosis [19].

Les méthodes bactériologiques à mettre en œuvre comprennent la recherche de bacilles acido-alcoolo-résistants (BAAR) par l’examen microscopique, la mise en culture sur milieux spécifiques, l’identification par méthode moléculaire ou biochimique des bacilles obtenus en culture et les tests de sensibilité aux antituberculeux [19]. La présence de granulomes épithélio-gigantocellulaires est un argument présomptif dans un contexte clinique évocateur. La nécrose caséeuse constitue la lésion spécifique. La bactériologie est nécessaire pour confirmer le diagnostic et éliminer, en particulier, une mycobactérie non tuberculeuse [17]. Le traitement de la tuberculose amygdalienne est essentiellement médical, basé sur la poly-chimiothérapie anti-bacillaire [7, 16]. L’amygdalectomie n’est pas systématique, elle dépend de l’aspect et du retentissement clinique mais aussi de la durée de la maladie [20].

Les antituberculeux utilisés sont au nombre de six. Les uns à la fois bactéricides, et stérilisants (HRSZ), les autres essentiellement bactériostatiques (Ethionamide, Ethambutol). Ce traitement peut être utilisé soit en régime court de 6 à 9mois [21] ou en régime classique de 12 mois [22]. Les objectifs de ce dernier sont d’abord la stérilisation du foyer tuberculeux grâce à l’action stérilisante de certains antituberculeux bactéricides (HRZ), mais aussi la prévention d’une résistance secondaire par l’association de plusieurs anti-bacillaires. Dans les formes disséminées, il est recommandé par l’Organisation Mondiale de la Santé de poursuivre une bithérapie (Rifadine et Isoniazide) pendant 7 à 10 mois après la quadrithérapie de deux mois [21].

L’évolution sous traitement est souvent favorable avec une disparition des lésions en quelques semaines [8, 23]. Le risque de rechute ou de nonguérison malgré un traitement bien conduit est de 1 % [21]. Ces échecs sont dus à l’apparition de souches résistantes du BK aux anti-bacillaires [24].

## Conclusion

Les localisations de la tuberculose sont variées et ses aspects cliniques sont trompeurs. Son diagnostic peut constituer un véritable challenge pour le clinicien qui doit pouvoir le relever vue la recrudescence actuelle de la maladie.

## Conflits d’intérêts

Les auteurs déclarent n’avoir aucun conflit d’intérêt.
